# Dynamic contrast-enhanced MRI deep learning model combined with clinical model for noninvasive differentiation of hepatocellular carcinoma from mass-forming intrahepatic cholangiocarcinoma

**DOI:** 10.3389/fonc.2026.1936989

**Published:** 2026-10-23

**Authors:** Huijie Zhang, Lijuan Guo, Fei Jia, Wenling Liu, Meng Zhang, Feng Li, Jun Zhou, Baolin Wu

**Affiliations:** 1 Department of Medical Imaging, Xinxiang Central Hospital, Xinxiang, China; 2 Department of Nuclear Medicine, The First Affiliated Hospital of Henan Medical University, Weihui, China; 3 Department of Magnetic Resonance, The Second Hospital and Clinical Medical School, Lanzhou University, Lanzhou, China; 4 Gansu Province Clinical Research Center for Functional and Molecular Imaging, Lanzhou, China; 5 Gansu Medical MRI Equipment Application Industry Technology Center, Lanzhou, China; 6 Department of Magnetic Resonance, The First Affiliated Hospital of Henan Medical University, Weihui, China; 7 Interventional Diagnostic and Therapeutic Center, Zhongnan Hospital of Wuhan University, Wuhan, China; 8 Department of Radiology, Huaxi MR Research Center (HMRRC), Institute of Radiology and Medical Imaging, West China Hospital of Sichuan University, Chengdu, China; 9 Psychoradiology Key Laboratory of Sichuan Province, West China Hospital of Sichuan University, Chengdu, China

**Keywords:** deep learning, dynamic contrast-enhanced magnetic resonance imaging, hepatocellular carcinoma, mass-forming intrahepatic cholangiocarcinoma, multiphase fusion

## Abstract

**Rationale and objectives:**

This study aims to develop a framework integrating dynamic contrast-enhanced magnetic resonance imaging (DCE-MRI)-based multi-channel deep learning combined with clinical modeling for the non-invasive preoperative discrimination between hepatocellular carcinoma (HCC) and mass-forming intrahepatic cholangiocarcinoma (MF-ICC).

**Methods:**

The internal development cohort comprised 334 surgically confirmed patients, including 231 patients with HCC and 103 with MF-ICC, and was divided at the patient level into a training cohort (*n* = 233) and an internal validation cohort (*n* = 101). The independent external validation cohort included 50 patients, comprising 40 with HCC and 10 with MF-ICC. We constructed separate two-dimensional deep learning models based on arterial phase, portal venous phase, and delayed phase images and then developed fusion models (2D model). A multi-channel deep learning model was further developed by directly inputting the three-phase images stacked along the channel dimension. Moreover, a clinical model was built based on independent predictors identified through multivariate analysis, which was ultimately integrated with the best-performing deep learning model. Model performance was evaluated and compared.

**Results:**

Deep learning model directly feeding the three-phase images as a multi-channel input achieved better discrimination (area under the curve [AUC] = 0.814 in the internal validation cohort) than all single-phase and corresponding fusion models. The comprehensive 2D + multi-channel + clinical model showed the best performance for the differentiation of HCC from MF-ICC, achieving an AUC of 0.887 (95% CI, 0.819–0.954) in the internal validation cohort, and generalized well to the external validation cohort (AUC = 0.908; 95% CI, 0.811–1.000). However, no significant differences in AUCs were found among the models after correction for multiple comparisons.

**Conclusion:**

The fusion model integrating clinical predictors with DCE-MRI multi-phase deep learning feature may provide a valuable tool for preoperatively differentiating HCC from MF-ICC.

## Introduction

Intrahepatic cholangiocarcinoma (ICC) is a primary malignant tumor of the liver. Among its subtypes, mass-forming intrahepatic cholangiocarcinoma (MF-ICC) constitutes approximately 60% of all ICC cases and represents the second most common primary liver malignancy after hepatocellular carcinoma (HCC) (1). MF-ICC and HCC share several common risk factors, including cirrhosis and chronic viral hepatitis (2). However, their treatment strategies differ substantially: surgical resection offers the only potential cure for MF-ICC (1), whereas HCC management encompasses multiple options such as percutaneous ablation, radiofrequency ablation, surgical resection, and liver transplantation (3, 4). Consequently, achieving accurate preoperative discrimination between MF-ICC and HCC is of paramount clinical significance.

Currently, preoperative differential diagnosis relies primarily on conventional imaging examinations, such as dynamic contrast-enhanced magnetic resonance imaging (DCE-MRI) (5). However, substantial overlap exists in the imaging appearances of MF-ICC and HCC—for instance, some infiltrative HCCs can exhibit imaging features similar to those of MF-ICC (2). This overlap poses a significant challenge to traditional imaging diagnosis, which depends heavily on the subjective experience of radiologists.

To address these limitations, quantitative analysis methods based on medical imaging have emerged. Traditional radiomics approaches, which extract handcrafted quantitative features, have demonstrated certain potential in tumor differentiation (6). However, these traditional machine learning methods often struggle to fully capture the complex information inherent in multi-phase DCE-MRI data. In contrast, deep learning models have shown remarkable advantages due to their ability to automatically learn and extract complex features from large-scale datasets (7). Nevertheless, existing studies are mostly limited to single-phase analysis, failing to fully utilize the comprehensive hemodynamic information provided by multi-phase enhancement (8). Notably, the multi-channel deep learning architecture, by processing different images as parallel inputs, enables models to synergistically analyze features across different images, thereby capturing comprehensive tumor information (9). This technique provides a novel technological pathway for accurately discriminating MF-ICC from HCC, potentially overcoming the diagnostic bottlenecks of conventional methods.

This study aimed to compare several approaches for integrating multiphase DCE-MRI information for the differentiation of HCC from MF-ICC. Specifically, we developed single-phase 2D models, decision-level stacking and arithmetic ensemble models, and a phase-stacked multi-channel model using spatially matched multi-phase maps of DCE-MRI. We further evaluated whether integrating strictly preoperative clinical and semantic imaging predictors with the prespecified imaging models provided incremental diagnostic information. The robustness and reproducibility of the developed models were validated in an independent external cohort.

## Materials and methods

### Study design and patient cohorts

This retrospective study included an internal development cohort and an independent external validation cohort. A STARD-compatible patient-selection flow diagram is presented in **Figure 1**. For the internal cohort, consecutive patients with suspected primary malignant liver tumors who underwent preoperative DCE-MRI between September 2016 and June 2024 were assessed for eligibility. The pathological diagnoses of HCC and MF-ICC were independently confirmed by two senior pathologists using surgical resection specimens. The inclusion criteria were (1) histopathologically confirmed HCC or MF-ICC after surgical resection (2), preoperative DCE-MRI performed within 1 month before surgery, and (3) a maximum lesion diameter greater than 1.0 cm. The exclusion criteria were (1) previous antitumor therapy, including transarterial chemoembolization, radiofrequency ablation, immunotherapy, or systemic treatment (2), macrovascular invasion, extrahepatic metastasis, or another concurrent malignancy (3), poor image quality, including inadequate interphase registration (4), incomplete clinical, pathological, or imaging data, and (5) mixed HCC-ICC or another pathological tumor type. Of 508 patients assessed at the internal dataset, 174 were excluded, and 334 patients were included in the internal development cohort. The internal cohort comprised 231 patients with HCC and 103 with MF-ICC. Only the largest pathologically confirmed lesion was analyzed for each patient. The cohort was divided at the patient level using stratified random sampling into a training cohort of 233 patients and an internal validation cohort of 101 patients. All images and derived data from an individual patient were assigned exclusively to one cohort.

**Figure 1:**
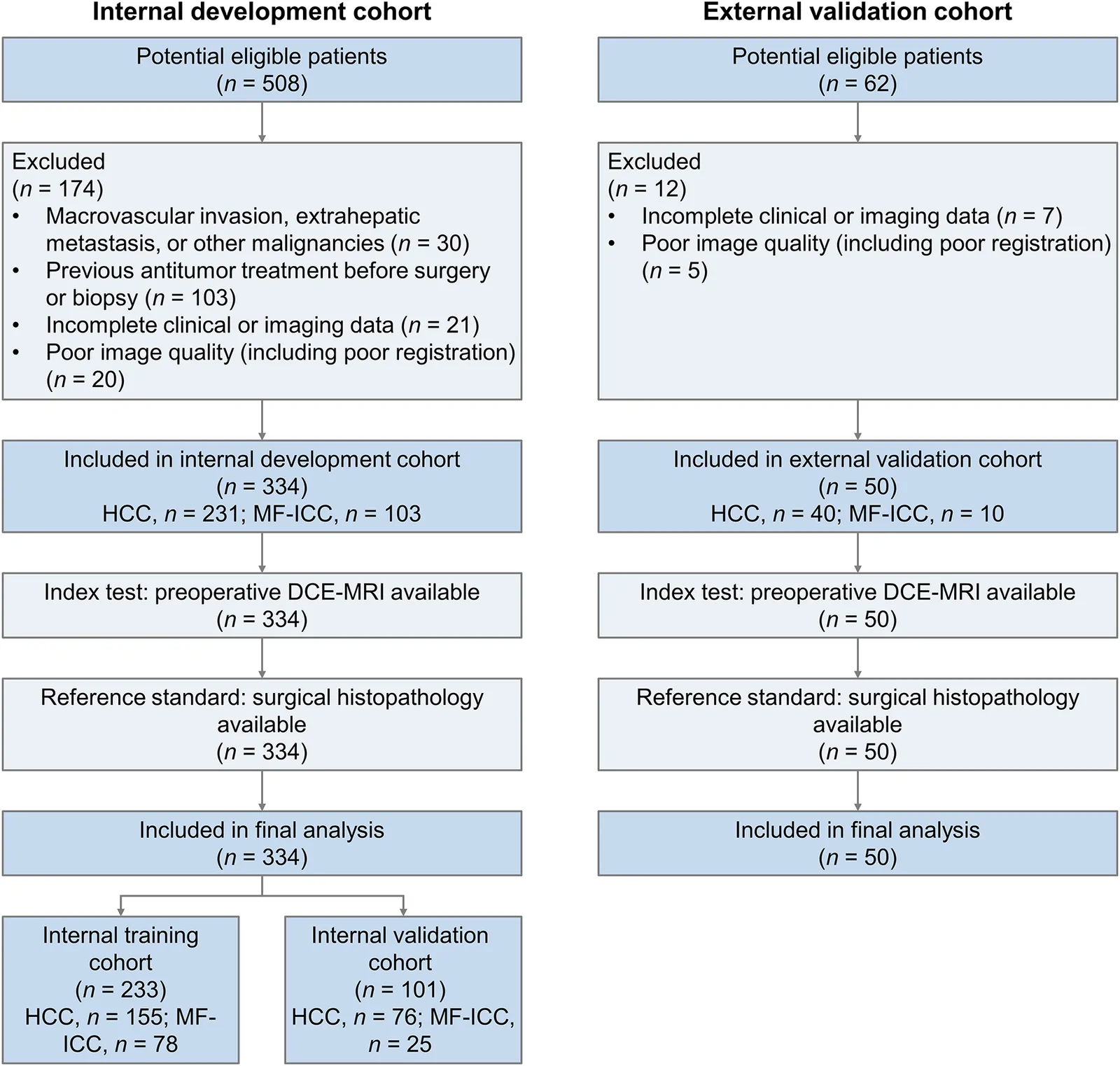
Flowchart of patient recruitment and cohort allocation. HCC, hepatocellular carcinoma; MF-ICC, mass-forming intrahepatic cholangiocarcinoma.

For the external validation cohort, consecutive patients were retrospectively enrolled at the collaborating institution between September 2023 and June 2025 using the same eligibility criteria. Of the 62 patients assessed, 12 were excluded because of poor image quality (*n* = 5) or incomplete clinical or imaging data (*n* = 7), leaving a final cohort of 50 patients, including 40 with HCC and 10 with MF-ICC.

The internal study was approved by the institutional ethics committee of Xinxiang Central Hospital and Fourth Clinical College of Henan Medical University (approval no. 2022-181), and the requirement for informed consent was waived. The external institution provided fully anonymized historical data under an ethics waiver, and no identifiable patient information was transferred.

### Demographic, laboratory, and semantic imaging variables

Demographic and preoperative clinical variables, including age, sex, hepatitis B surface antigen status, alpha-fetoprotein level, cirrhosis status, and maximum tumor diameter, were retrieved from electronic medical records. Laboratory tests were performed within 1 week before treatment. Postoperative immunohistochemical markers, including cytokeratin 19 and Ki-67, were used solely for descriptive characterization of the cohorts and were not incorporated into the clinical–radiological or any fusion model.

Semantic imaging features were assessed by a radiologist with 7 years of abdominal imaging experience who was blinded to the pathological diagnosis. Enhancing capsule was evaluated on portal venous phase (PVP) images and defined as a smooth and uniform enhancing rim surrounding the lesion. Arterial-phase (AP) rim enhancement was evaluated on AP images and defined as continuous or discontinuous peripheral hyperenhancement. Peritumoral enhancement was evaluated on AP and PVP images and defined as enhancement of the liver parenchyma immediately adjacent to the tumor margin. Tumor margin was assessed across all phases and categorized as smooth or non-smooth. Internal arteries were evaluated on AP images and defined as visible intratumoral arterial branches.

### MRI acquisition

For the internal dataset, DCE-MRI examinations were performed using a 3.0-T Philips Ingenia scanner with an eight-channel phased-array body coil combined with spine coil elements. Gadobutrol (Gadavist) was administered intravenously at a dose of 0.1 mmol/kg and a rate of 2.0 mL/s using a power injector, followed by a 20-mL saline flush. The DCE-MRI protocol included unenhanced imaging followed by AP, PVP, and delayed phase (DP) acquisitions. AP images were acquired 20–30 s after contrast injection, PVP images at 60–70 s, and DP images at 3–5 min. A fat-suppressed three-dimensional T1-weighted gradient-echo THRIVE sequence was used with the following principal parameters: repetition time/echo time, 3.0/1.2 ms; flip angle, 15°; field of view, 380 × 380 mm; matrix, 128 × 128; slice thickness, 6 mm; interslice gap, 0 mm; and 20 slices covering the entire liver.

For the external dataset, DCE-MRI examinations were performed using a 3.0-T GE Healthcare MRI system with a fat-suppressed three-dimensional T1-weighted gradient-echo LAVA sequence. The contrast agent, dose, injection rate, saline flush, phase-acquisition times, and principal spatial parameters were consistent with those used in the internal cohort.

### Image preprocessing

Image preprocessing included N4 bias-field correction, resampling to an isotropic voxel size of 1 × 1 × 1 mm³, and rigid registration of the PVP and DP images to the AP image. Registration quality was independently inspected by two radiologists. Three cases with inadequate interphase registration were included among the 20 internal patients excluded for poor image quality. For model input, grayscale values were normalized using ImageNet statistics. Single-phase grayscale images were replicated across three channels before normalization using mean values of 0.485, 0.456, and 0.406, respectively, and standard deviation values of 0.229, 0.224, and 0.225, respectively.

### Tumor segmentation and image selection

A radiologist with 7 years of abdominal imaging experience manually delineated three-dimensional volumes of interest encompassing the entire tumor on each phase using ITK-SNAP. All segmentations were reviewed and confirmed by a senior radiologist with 15 years of experience. To evaluate interobserver reproducibility, a random subset of 30 internal cases was independently segmented by a second radiologist with 8 years of experience. The interobserver Dice similarity coefficient was 0.91 ± 0.04. For model development, the axial slice containing the largest tumor cross-sectional area was selected. For the single-phase models, the corresponding largest-section image was selected separately from each phase. For the phase-stacked multi-channel model, the slice showing the largest tumor area was identified on the AP image, and the spatially corresponding PVP and DP slices were retrieved after registration.

### Evaluation of clinical predictors

Candidate preoperative clinical predictors were evaluated in the training cohort using logistic regression analysis. Variables with a univariable *p*-value <0.05 were entered into a multivariable logistic regression model with backward stepwise elimination. MF-ICC was coded as 1 and HCC as 0. Variables retained at *p <*0.05 in the final multivariable model constituted the clinical predictor set. Variance inflation factors were calculated for the retained predictors to assess multicollinearity.

### Single-phase 2D models and decision-level fusion

A rectangular bounding box tightly enclosing the tumor was extracted from the selected slice and resized to 256 × 256 pixels using bilinear interpolation. Each patient contributed one image per phase, and neither patch extraction nor patient-level aggregation was performed. Separate AP, PVP, and DP classifiers were developed. The backbone architecture was VGG11 with batch normalization (VGG11-bn), randomly initialized without ImageNet-pretrained weights. The original classifier was replaced with global average pooling, a fully connected layer of 256 units with rectified linear unit (ReLU) activation, a dropout layer (rate 0.5), and a final sigmoid-activated output neuron.

During training, random horizontal and vertical flips were applied to the single-phase models. Then, two decision-level fusion approaches were evaluated. In the stacking approach, patient-level probabilities generated by the AP, PVP, and DP models served as meta-features. Fivefold cross-validation within the training cohort was used to generate out-of-fold probabilities, which were then used to train a logistic-regression meta-classifier. This prespecified stacking-based decision-level fusion model is referred to as the 2D model. In the arithmetic ensemble fusion, the predicted probabilities from the AP, PVP, and DP models were averaged without fitting an additional classifier. The stacking-based 2D model had been prespecified for the downstream clinical–radiological fusion before the external validation results were examined; it was not selected on the basis of its highest internal-validation AUC.

### Phase-stacked multi-channel model

A phase-stacked multi-channel model was developed to process spatially matched AP, PVP, and DP images simultaneously. A minimal bounding rectangle minimally encompassing the tumor across all three registered phases was applied identically to each image. Each cropped image was resized to 256 × 256 pixels, and the three single-channel phase images were stacked along the channel dimension to form a 256 × 256 × 3 input tensor. The model used the same VGG11-bn backbone and classification head as the single-phase models, and the standard three-channel first convolutional layer was retained without modification. Random cropping, horizontal flipping, and vertical flipping were applied during training. All geometric transformations were synchronized across the AP, PVP, and DP channels to preserve spatial correspondence. The multi-channel pipeline, therefore, included random cropping in addition to the augmentation procedures used for the single-phase models.

Gradient-weighted class activation mapping (Grad-CAM) was generated for cases of the phase-stacked multi-channel model. Heatmaps were used qualitatively to assess whether model attention was concentrated within the tumor rather than in the background or extratumoral tissue. **Figure 2** shows representative Grad-CAM maps. Notably, attention was generally concentrated within tumor-containing image regions rather than in background structures.

**Figure 2:**
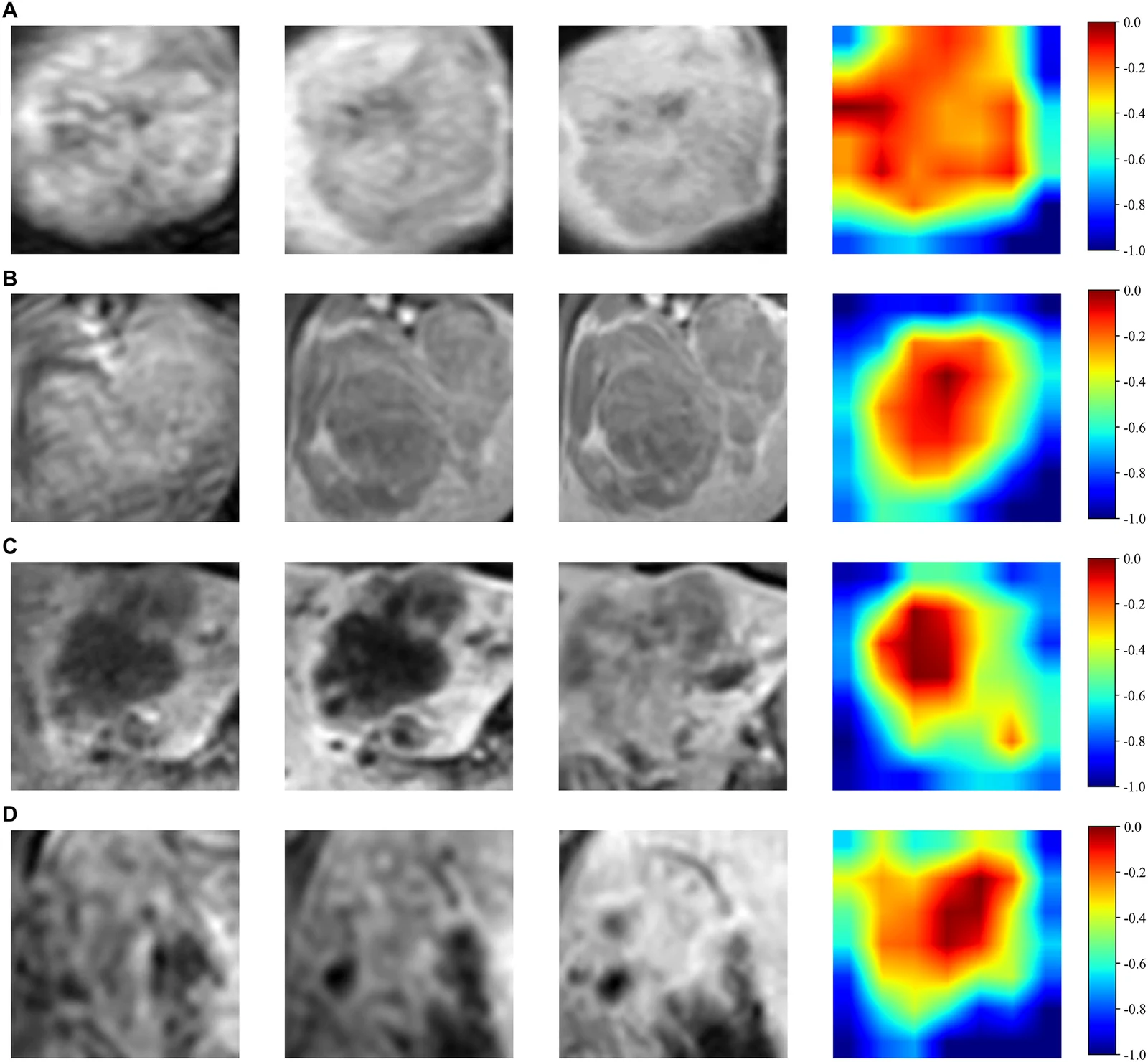
Representative Grad-CAM visualizations from the phase-stacked multi-channel model. For each row, the first three panels show arterial, portal venous, and delayed phase images, respectively, and the final panel shows the corresponding activation map. **(A, B)** represent HCC; **(C, D)** represent MF-ICC. The maps provide qualitative localization of model attention and should not be interpreted as mechanistic explanations.

### Fusion models

Based on the significant clinical predictors, 2D model, and multi-channel model, three fusion models were evaluated (1): the 2D model + the clinical predictors (2), the multi-channel model + the clinical predictors, and (3) the combination of the 2D model, multi-channel model, and clinical predictors. Within the training cohort, fivefold out-of-fold deep-learning probabilities were combined with the clinical–radiological predictors to train logistic-regression fusion classifiers. Neither internal-validation nor external-validation predictions were used to fit the fusion models.

### Model training and checkpoint selection

All deep-learning models were implemented using PyTorch version 2.0 and trained on an NVIDIA GeForce RTX 4060 GPU. Binary cross-entropy was used as the loss function. Optimization was performed using stochastic gradient descent with an initial learning rate of 0.01, momentum of 0.9, weight decay of 1 × 10^−4^, and a batch size of 32. A StepLR scheduler reduced the learning rate by a factor of 0.1 every 10 epochs. All deep-learning models were trained for 30 epochs. The checkpoint achieving the highest AUC in the internal validation cohort was retained for external evaluation, and no early-termination rule was applied. Neither class weighting nor oversampling was used. Default stochastic operations were used, and no explicit random seed was manually specified. Because the single-phase and multi-channel pipelines did not use completely identical augmentation procedures, differences in their performance cannot be attributed solely to the input-fusion strategy.

### Statistical analysis

Continuous variables were assessed for normality using the Shapiro–Wilk test. Normally distributed variables were compared using the independent-samples *t*-test, whereas non-normally distributed variables were compared using the Mann–Whitney *U*-test. Categorical variables were compared using the chi-square test or Fisher’s exact test, as appropriate. Complete-case analysis was used because missing data accounted for less than 5% of all included preoperative candidate predictors.

Model performance was evaluated using accuracy, area under the receiver operating characteristic (AUC) with 95% confidence interval (CI), sensitivity, specificity, positive predictive value (PPV), and negative predictive value (NPV). Classification thresholds were determined exclusively in the internal validation cohort by maximizing the Youden index. The selected threshold for each model was then fixed and applied unchanged to the external validation cohort. Differences in AUCs among different models were assessed using DeLong tests, with Bonferroni correction for multiple comparisons.

Calibration was assessed separately in the training, internal validation, and external validation cohorts using the Brier score, calibration intercept, calibration slope, and calibration plots. Stratified nonparametric bootstrapping with 2,000 resamples was used to estimate 95% CIs for calibration measures. Non-convergent bootstrap samples were omitted. Additionally, decision-curve analysis (DCA) was performed separately in each cohort over the prespecified threshold-probability range of 0–1.0. A positive model classification was interpreted as prompting consideration of an MF-ICC-oriented diagnostic, surgical-planning, or multidisciplinary-review pathway rather than directly determining treatment.

All statistical tests were two-sided and were performed using R and Python version 3.7.

## Results

### Patient selection and cohort characteristics

**Table 1** presents the clinical–radiological characteristics of the internal training, internal validation, and external validation cohorts. Comparisons of clinical–radiological characteristics between HCC and MF-ICC patients across the three cohorts are provided in **Supplementary Tables S1**-**S3**. Significant differences in several clinical variables were found between HCC and MF-ICC patients. Specifically, tumor diameter and arterial-phase rim enhancement differed between HCC and MF-ICC patients across all three cohorts. Sex, alpha-fetoprotein level, and intratumoral necrosis differed between the two patient groups in the training cohort, whereas cirrhosis differed in the external validation cohort. CK19 and Ki-67 were reported only as postoperative descriptive characteristics and were not used in model development.

**Table 1:** Demographic and clinical characteristics of patients in the internal training, internal validation, and external validation cohorts.

Variables	Internal training cohort (*n* = 233)	Internal validation cohort (*n* = 101)	*p*-value^a^	External validation cohort (*n* = 50)	*p*-value^b^
Age (year)	56.75 ± 9.27	54.95 ± 10.22	0.158	54.44 ± 11.49	0.205
Sex, *n* (%)			0.848		0.744
0, female	64 (27.47)	26 (25.74)		12 (24.00)	
1, male	169 (72.53)	75 (74.26)		38 (76.00)	
Ki-67 (%)	36.55 ± 21.80	38.04 ± 20.25	0.419	29.98 ± 20.21	0.054
CK19, *n* (%)			0.932		0.766
0, negative	168 (72.10)	74 (73.27)		35 (70.00)	
1, positive	65 (27.90)	27 (26.73)		15 (30.00)	
Tumor diameter, *n* (%)			0.318		0.015
0, <5 cm	116 (49.79)	57 (56.44)		35 (70.00)	
1, ≥5 cm	117 (50.21)	44 (43.56)		15 (30.00)	
Alpha-fetoprotein, *n* (%)			0.318		0.021
0, <400 ng/mL	149 (63.95)	71 (70.30)		41 (82.00)	
1, ≥400 ng/mL	84 (36.05)	30 (29.70)		9 (18.00)	
Hepatitis B surface antigen, *n* (%)			0.744		0.338
0, negative	30 (12.88)	11 (10.89)		4 (8.00)	
1, positive	203 (87.12)	90 (89.11)		46 (92.00)	
Cirrhosis, *n* (%)			0.714		0.660
0, absent	63 (27.04)	30 (29.70)		12 (24.00)	
1, present	170 (72.96)	71 (70.30)		38 (76.00)	
Intratumoral necrosis, *n* (%)			1.000		0.672
0, absent	38 (16.31)	16 (15.84)		10 (20.00)	
1, present	195 (83.69)	85 (84.16)		40 (80.00)	
Tumor margin, *n* (%)			0.645		1.000
0, smooth	89 (38.20)	42 (41.58)		19 (38.00)	
1, non-smooth	144 (61.80)	59 (58.42)		31 (62.00)	
Internal arteries, *n* (%)			0.247		0.825
0, absent	133 (57.08)	50 (49.50)		30 (60.00)	
1, present	100 (42.92)	51 (50.50)		20 (40.00)	
Peritumoral enhancement, *n* (%)			0.153		0.072
0, absent	124 (53.22)	63 (62.38)		19 (38.00)	
1, present	109 (46.78)	38 (37.62)		31 (62.00)	
Enhancing capsule, *n* (%)			0.256		0.003
0, absent	89 (38.20)	46 (45.54)		31 (62.00)	
1, present	144 (61.80)	55 (54.46)		19 (38.00)	
Arterial-phase rim enhancement, *n* (%)			0.438		0.590
0, absent	152 (65.24)	71 (70.30)		30 (60.00)	
1, present	81 (34.76)	30 (29.70)		20 (40.00)	

^a^ Comparison between the training and internal validation cohorts.

^b^ Comparison between the training and external validation cohorts.

### Performance of the clinical model

As shown in **Table 2**, univariable analysis identified sex, tumor diameter, AFP, intratumoral necrosis, and arterial-phase rim enhancement as candidate predictors (all *p* < 0.05). Multivariable logistic regression suggested that tumor diameter ≥5 cm (OR, 17.762; 95% CI, 6.999–45.077; *p* < 0.001), AFP ≥400 ng/mL (OR, 3.633; 95% CI, 1.650–8.003; *p* = 0.001), and arterial-phase rim enhancement (OR, 15.380; 95% CI, 6.555–36.085; *p* < 0.001) were independently associated with MF-ICC. The final model intercept was −4.177, and all retained-predictor variance inflation factors were below 1.10, which was substantially lower than the threshold of 5, indicating no substantial multicollinearity among predictors. The clinical–radiological model achieved an AUC of 0.784 (95% CI, 0.672–0.895) and 0.745 (95% CI, 0.547–0.943) in the internal validation cohort and external validation cohort, respectively. Confusion matrix and classification thresholds are shown in **Supplementary Tables S4**, **S5**, respectively.

**Table 2:** Univariable and multivariable logistic regression analyses of preoperative predictors for MF-ICC.

Clinical variables	Univariable analysis		Multivariable analysis	
	OR (95% CI)	*p*-value	OR (95% CI)	*p*-value
Sex (male vs. female)	0.450 (0.248–0.814)	0.008	–	–
Age (per year)	1.010 (0.981–1.041)	0.506	–	–
Tumor diameter (≥5 cm vs. <5 cm)	14.710 (6.982–30.991)	<0.001	17.762 (6.999–45.077)	<0.001
AFP (≥400 vs. <400 ng/mL)	3.721 (2.095–6.607)	<0.001	3.633 (1.650–8.003)	0.001
HBsAg (positive vs. negative)	0.723 (0.329–1.588)	0.419	–	–
Cirrhosis (present vs. absent)	1.231 (0.660–2.297)	0.514	–	–
Intratumoral necrosis (present vs. absent)	0.381 (0.188–0.773)	0.007	–	–
Tumor margin (non-smooth vs. smooth)	0.771 (0.442–1.345)	0.360	–	–
Internal arteries (present vs. absent)	1.425 (0.824–2.465)	0.205	–	–
Peritumoral enhancement (present vs. absent)	1.418 (0.821–2.448)	0.210	–	–
Enhancing capsule (present vs. absent)	1.370 (0.775–2.421)	0.279	–	–
Arterial-phase rim enhancement (present vs. absent)	11.865 (6.233–22.585)	<0.001	15.380 (6.555–36.085)	<0.001

MF-ICC was coded as 1 and HCC as 0. Univariable and multivariable logistic regression analyses were performed in the training cohort. The final model intercept was −4.177.

OR, odds ratio; CI, confidence interval; AFP, alpha-fetoprotein; HBsAg, hepatitis B surface antigen.

### Performance of the single-phase and decision-level fusion models

The performance, confusion matrices, and classification thresholds of the single-phase and decision-level fusion models are shown in **Table 3** and in **Supplementary Tables S4**, **S5**, respectively. The stacking-based 2D model achieved the highest AUC of 0.849 in the training cohort. In the internal and external validation cohorts, the five models showed comparable performance for differentiation of HCC from MF-ICC.

**Table 3:** Diagnostic performance of the single-phase and decision-level fusion models.

Models	AUC (95% CI)	Accuracy	Sensitivity	Specificity	PPV	NPV
Internal training cohort						
AP	0.751 (0.683–0.818)	0.747	0.590	0.826	0.630	0.800
DP	0.717 (0.646–0.787)	0.652	0.744	0.606	0.487	0.825
PVP	0.741 (0.674–0.808)	0.700	0.679	0.710	0.541	0.815
Stacking	0.849 (0.798–0.901)	0.785	0.756	0.800	0.656	0.867
Ensemble	0.772 (0.708–0.836)	0.725	0.692	0.742	0.574	0.827
Internal validation cohort						
AP	0.768 (0.666–0.871)	0.752	0.720	0.763	0.500	0.892
DP	0.764 (0.650–0.878)	0.842	0.480	0.961	0.800	0.849
PVP	0.789 (0.683–0.895)	0.752	0.680	0.776	0.500	0.881
Stacking	0.754 (0.635–0.874)	0.752	0.640	0.789	0.500	0.870
Ensemble	0.779 (0.676–0.883)	0.723	0.760	0.711	0.463	0.900
External validation cohort						
AP	0.925 (0.852–0.998)	0.860	1.000	0.825	0.588	1.000
DP	0.887 (0.786–0.989)	0.780	0.900	0.750	0.474	0.968
PVP	0.925 (0.852–0.998)	0.840	1.000	0.800	0.556	1.000
Stacking	0.920 (0.831–1.000)	0.860	0.900	0.850	0.600	0.971
Ensemble	0.948 (0.891–1.000)	0.900	1.000	0.875	0.667	1.000

MF-ICC was treated as the positive class.

AP, arterial phase; PVP, portal venous phase; DP, delayed phase; 2D, prespecified stacking-based decision-level fusion of the AP, PVP, and DP models; Ensemble, arithmetic mean of the AP, PVP, and DP predicted probabilities; AUC, area under the receiver operating characteristic curve; CI, confidence interval; PPV, positive predictive value; NPV, negative predictive value.

### Performance of the fusion models

**Figure 3** and **Table 4** show the performance of the six prespecified principal models. The comprehensive 2D + multi-channel + clinical model showed the best performance for the differentiation of HCC from MF-ICC, achieving an AUC of 0.887 (95% CI, 0.819–0.954) in the internal validation cohort and generalized well to the external validation cohort (AUC = 0.908; 95% CI, 0.811–1.000). Confusion matrix and classification thresholds are shown in **Supplementary Tables S4**, **S5**, respectively.

**Figure 3:**
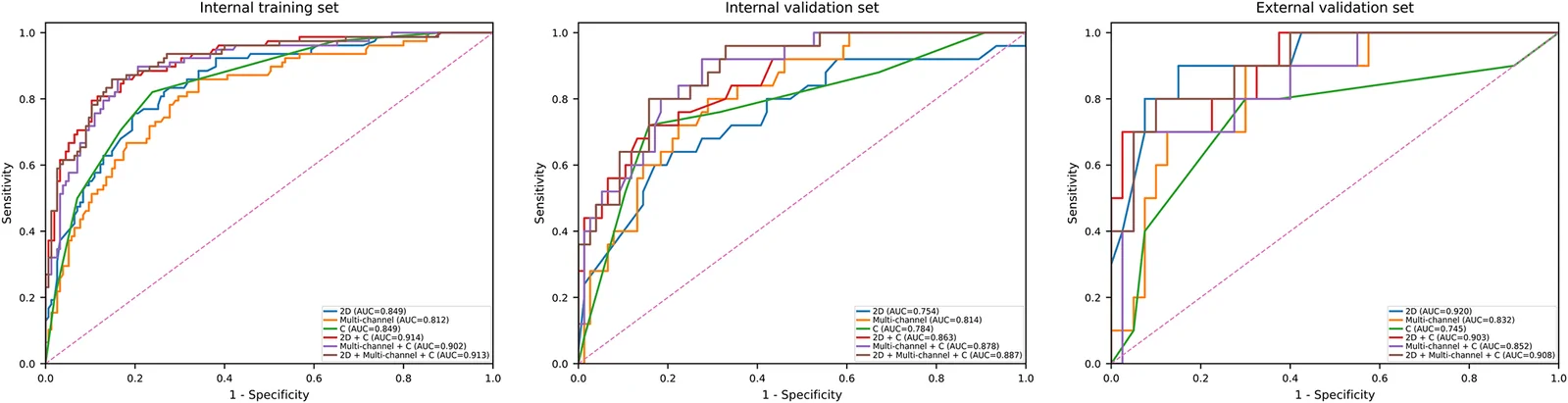
Receiver operating characteristic curves of the six prespecified principal models in the internal training, internal validation, and external validation cohorts.

**Table 4:** Diagnostic performance of the six principal models.

Models	AUC (95% CI)	Accuracy	Sensitivity	Specificity	PPV	NPV
Internal training cohort						
2D	0.849 (0.798-0.901)	0.785	0.756	0.800	0.656	0.867
Multi-channel	0.812 (0.754-0.871)	0.725	0.859	0.658	0.558	0.903
Clinical	0.849 (0.799-0.899)	0.781	0.821	0.761	0.634	0.894
2D + C	0.914 (0.875-0.953)	0.863	0.795	0.897	0.795	0.897
Multi-channel + C	0.902 (0.860-0.944)	0.845	0.859	0.839	0.728	0.922
2D + multi-channel + C	0.913 (0.874-0.953)	0.854	0.859	0.852	0.744	0.923
Internal validation cohort						
2D	0.754 (0.635-0.874)	0.752	0.640	0.789	0.500	0.870
Multi-channel	0.814 (0.725-0.903)	0.733	0.800	0.711	0.476	0.915
Clinical	0.784 (0.672-0.895)	0.812	0.720	0.842	0.600	0.901
2D + C	0.863 (0.784-0.941)	0.812	0.720	0.842	0.600	0.901
Multi-channel + C	0.878 (0.808-0.949)	0.772	0.920	0.724	0.523	0.965
2D + multi-channel + C	0.887 (0.819-0.954)	0.832	0.800	0.842	0.625	0.928
External validation cohort						
2D	0.920 (0.830-1.000)	0.860	0.900	0.850	0.600	0.971
Multi-channel	0.832 (0.701-0.964)	0.740	0.900	0.700	0.429	0.966
Clinical	0.745 (0.547-0.943)	0.720	0.800	0.700	0.400	0.933
2D + C	0.903 (0.800-1.000)	0.920	0.700	0.975	0.875	0.929
Multi-channel + C	0.852 (0.719-0.986)	0.900	0.700	0.950	0.778	0.927
2D + multi-channel + C	0.908 (0.811-1.000)	0.880	0.800	0.900	0.667	0.947

MF-ICC was treated as the positive class.

2D, prespecified stacking-based decision-level fusion model; C, clinical model; AUC, area under the curve; CI, confidence interval; PPV, positive predictive value; NPV, negative predictive value.

In the internal validation cohort, the 2D + clinical model differed significantly from the clinical model (*p* = 0.006, uncorrected). The multi-channel + clinical model differed significantly from both the 2D model (*p* = 0.049, uncorrected) and the clinical model (*p* = 0.027, uncorrected). The comprehensive model differed significantly from the multi-channel model (*p* = 0.021, uncorrected) and the 2D model (*p* = 0.010, uncorrected). In the external validation cohort, the 2D + clinical model differed significantly from both the clinical model (*p* = 0.011, uncorrected) and the multi-channel + clinical model (*p* = 0.032, uncorrected). However, none of these differences remained significant after Bonferroni correction (**Supplementary Table S6**).

### Calibration and decision-curve analysis results

The calibration results are presented in **Figure 4A**; **Supplementary Table S7**. In the internal validation cohort, the lowest Brier scores were observed for the multi-channel + clinical model (0.129), the comprehensive model (0.133), and the 2D + clinical model (0.138). Their calibration slopes were 2.023, 2.693, and 2.284, respectively, indicating that the predicted probabilities were insufficiently dispersed. The corresponding calibration intercepts were −0.232, 0.115, and 0.025. Overall, the calibration curves showed variable agreement between predicted probabilities and observed outcomes, and the findings do not support a general claim of good calibration.

**Figure 4:**
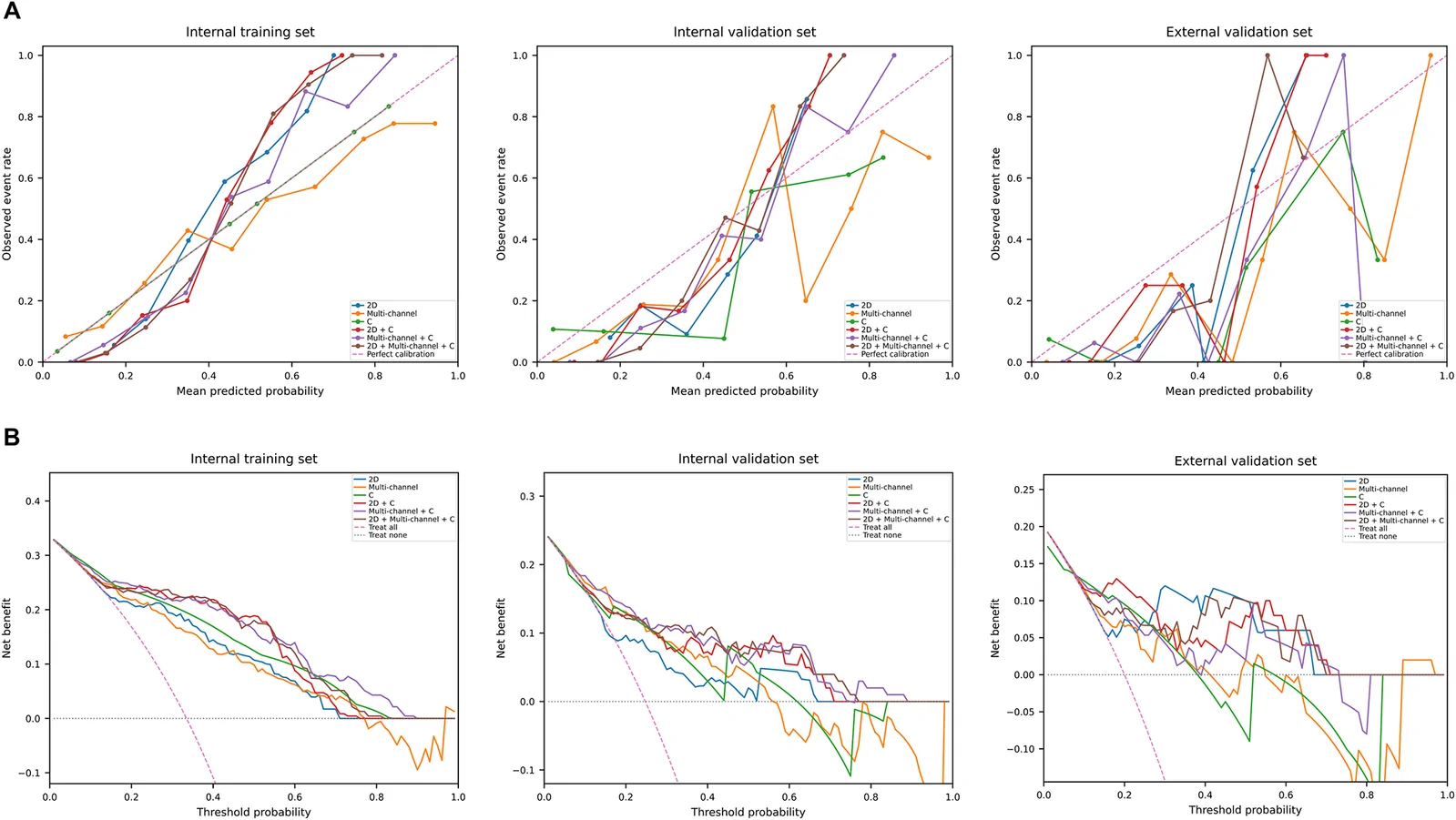
Exploratory calibration and decision-curve analyses of the six principal models in the internal training, internal validation, and external validation cohorts. **(A)** Calibration curves. The diagonal line represents perfect calibration. **(B)** Decision-curve analysis over the prespecified threshold-probability range of 0–1.0. A positive classification represents consideration of an MF-ICC-oriented diagnostic, surgical-planning, or multidisciplinary-review pathway.

As shown in **Figure 4B**, in the internal validation cohort, DCA showed that the three fusion models provided positive net benefit relative to the treat-all and treat-none strategies across most of the prespecified threshold-probability range of 0–1.0, whereas the individual 2D, multi-channel, and clinical models showed positive net benefit over narrower ranges. These findings were exploratory and depended on the clinical interpretation of the selected threshold probability.

## Discussion

This study proposed a novel multi-channel deep learning framework for distinguishing HCC from MF-ICC by simultaneously inputting AP, PVP, and DP images from DCE-MRI. Through further integrating clinical features with independent predictive value, the final fusion model demonstrated excellent discriminative performance. The findings indicate that the model combining multi-channel deep learning with clinical characteristics has potential diagnostic capability for the noninvasive preoperative differentiation between HCC and MF-ICC.

The most significant finding of this study is that the multi-channel deep learning model demonstrated substantially superior performance compared to conventional single-phase deep learning approaches. This advantage may be attributed to the inherent limitation of single-phase analysis, which captures only static enhancement features at specific time points, whereas the multi-channel architecture conceptualizes the enhancement process as a continuous dynamic entity. By enabling synchronous processing of multi-phase images at the initial convolutional layers, the model acquires the capacity to autonomously extract inter-phase correlated features (10–12). This methodology aligns with the approach demonstrated in a prior study, where stacking multiple b-value images along the channel dimension enabled the model to capture richer contextual features across different b-values (13). The innovative aspect of our research lies in the channel-wise stacking of multi-phase images, allowing the model to directly learn inherently cross-phase enhancement patterns, such as the typical “wash-in/wash-out” of HCC and the common “progressive delayed enhancement” of ICC. This approach not only overcomes the limitations of traditional radiomics that relies on handcrafted features and struggles to capture complex nonlinear relationships (14) but also explains why its performance surpasses that of models utilizing multi-phase information solely through decision-level fusion, as the latter fails to achieve synergistic integration at the feature level (15).

The performance of multi-channel deep learning model also outperformed that of the clinical model, indicating that deep learning features extracted from original multi-phase images possess better representational capacity than manually interpreted semantic features (such as “wash-in/wash-out” and “delayed enhancement”) (16). This phenomenon may be attributed to the substantial loss of subtle spatiotemporal information, imperceptible to the human eye, during the summarization process of semantic features, whereas deep learning models can learn complete hemodynamic patterns essential for differential diagnosis directly from pixel-level data without information loss (17). This finding further validates the unique value of deep learning in capturing complex spatiotemporal characteristics in medical imaging (18). The multi-channel approach demonstrates particular advantage in characterizing intratumor heterogeneity and capturing differential perfusion patterns between HCC and MF-ICC, which are often inadequately represented by conventional semantic descriptors.

Moreover, our study found that integrating the multi-channel deep learning model and the 2D deep learning model with clinical predictors could further enhance the diagnostic performance. This finding suggests that deep features and clinical indicators carry diagnostic information from different yet complementary dimensions. The deep imaging features extracted by the deep learning model from pixel-level data can reveal subtle anatomical patterns and hemodynamic heterogeneity that are difficult for human vision to discern, while clinical indicators provide crucial pathophysiological context at the level of molecular biology and background liver disease status (19). This integration essentially simulates the cognitive process of clinical experts in comprehensive diagnosis by synthesizing imaging manifestations with clinical history, thereby constructing a more robust and generalizable intelligent diagnostic system (20). Thus, this fusion model that we developed shows promise as a reliable computer-aided diagnostic tool in future clinical practice. Particularly when dealing with challenging cases with atypical or overlapping imaging presentations, it can provide objective decision support for radiologists, facilitate individualized treatment planning, and ultimately help improve patient outcomes.

Nevertheless, this study has several limitations that should be acknowledged. First, this is a retrospective study with a relatively small sample size; the model’s performance generalizability requires further validation through prospective, multi-center datasets. Second, despite implementing data augmentation techniques, expanding the sample size, particularly by incorporating more cases of rare MF-ICC subtypes, would enhance the model’s universality and robustness. Finally, the current tumor segmentation still requires manual completion, which may introduce variability. Future research should explore end-to-end joint models that integrate automatic segmentation and classification to further improve workflow automation.

In summary, this study has preliminarily demonstrated the advantage of a DCE-MRI-based multi-channel deep learning model for discriminating between HCC and MF-ICC. Furthermore, by integrating additional clinically relevant indicators, the final comprehensive model achieved the best diagnostic performance. This model may offer a noninvasive and efficient tool to support preoperative decision-making, helping clinicians stratify patients more accurately and potentially guiding individualized treatment planning.

## Data Availability

The original contributions presented in the study are included in the article/**Supplementary Material**. Further inquiries can be directed to the corresponding authors.
