# Supplementary material for: Dynamic contrast-enhanced MRI deep learning model combined with clinical model for noninvasive differentiation of hepatocellular carcinoma from mass-forming intrahepatic cholangiocarcinoma
Source: Front Oncol. 2026 Oct 23;16:1936989. doi: 10.3389/fonc.2026.1936989 (PMC13739590; doi:10.3389/fonc.2026.1936989)
Supplement: Supplementary file 1 [file Table1.docx]

**Dynamic contrast-enhanced MRI deep learning model combined with clinical model** **for noninvasive** **differentiation of hepatocellular carcinoma from mass-forming intrahepatic cholangiocarcinoma**

*Online Supplements*

**Contents**

**Supplementary Table S1.** Baseline characteristics of patients in the internal training cohort

**Supplementary Table S2.** Baseline characteristics of patients in the internal validation cohort

**Supplementary Table S3.** Baseline characteristics of patients in the external validation cohort

**Supplementary Table S4.** Confusion matrices of all models in the three cohorts

**Supplementary Table S5.** Classification thresholds selected in the internal validation cohort

**Supplementary Table S6.** Pairwise comparisons of AUCs among the six principal models

**Supplementary Table S7.** Exploratory calibration measures for the six principal models

**Table S1.** Baseline characteristics of patients in the internal training cohort

| **Variables** | **All (*n* = 233)** | **HCC (*n* = 155)** | **MF-ICC (*n* = 78)** | ***p* value** |
| --- | --- | --- | --- | --- |
| Age (year) | 56.75 ± 9.27 | 56.46 ± 8.87 | 57.32 ± 10.06 | 0.430 |
| Ki-67 (%) | 36.55 ± 21.80 | 34.83 ± 21.45 | 39.97 ± 22.21 | 0.076 |
| CK19, n (%) |  |  |  | <0.001 |
| -Negative | 168 (72.10) | 137 (88.39) | 31 (39.74) |  |
| -Positive | 65 (27.90) | 18 (11.61) | 47 (60.26) |  |
| Sex, n (%) |  |  |  | 0.012 |
| -Female | 64 (27.47) | 34 (21.94) | 30 (38.46) |  |
| -Male | 169 (72.53) | 121 (78.06) | 48 (61.54) |  |
| Tumor diameter, n (%) |  |  |  | <0.001 |
| -< 5 cm | 116 (49.79) | 106 (68.39) | 10 (12.82) |  |
| -≥ 5 cm | 117 (50.21) | 49 (31.61) | 68 (87.18) |  |
| Alpha-fetoprotein, n (%) |  |  |  | <0.001 |
| -< 400 ng/mL | 149 (63.95) | 115 (74.19) | 34 (43.59) |  |
| -≥ 400 ng/mL | 84 (36.05) | 40 (25.81) | 44 (56.41) |  |
| Hepatitis B surface antigen, n (%) |  |  |  | 0.546 |
| -Negative | 30 (12.88) | 18 (11.61) | 12 (15.38) |  |
| -Positive | 203 (87.12) | 137 (88.39) | 66 (84.62) |  |
| Cirrhosis, n (%) |  |  |  | 0.619 |
| -Absent | 63 (27.04) | 44 (28.39) | 19 (24.36) |  |
| -Present | 170 (72.96) | 111 (71.61) | 59 (75.64) |  |
| Intratumoral necrosis, n (%) |  |  |  | 0.011 |
| -Absent | 38 (16.31) | 18 (11.61) | 20 (25.64) |  |
| -Present | 195 (83.69) | 137 (88.39) | 58 (74.36) |  |
| Tumor margin, n (%) |  |  |  | 0.439 |
| -Smooth | 89 (38.20) | 56 (36.13) | 33 (42.31) |  |
| -Non-smooth | 144 (61.80) | 99 (63.87) | 45 (57.69) |  |
| Internal arteries, n (%) |  |  |  | 0.259 |
| -Absent | 133 (57.08) | 93 (60.00) | 40 (51.28) |  |
| -Present | 100 (42.92) | 62 (40.00) | 38 (48.72) |  |
| Peritumoral enhancement, n (%) |  |  |  | 0.264 |
| -Absent | 124 (53.22) | 87 (56.13) | 37 (47.44) |  |
| -Present | 109 (46.78) | 68 (43.87) | 41 (52.56) |  |
| Enhancing capsule, n (%) |  |  |  | 0.347 |
| -Absent | 89 (38.20) | 63 (40.65) | 26 (33.33) |  |
| -Present | 144 (61.80) | 92 (59.35) | 52 (66.67) |  |
| Arterial-phase rim enhancement, n (%) |  |  |  | <0.001 |
| -Absent | 152 (65.24) | 129 (83.23) | 23 (29.49) |  |
| -Present | 81 (34.76) | 26 (16.77) | 55 (70.51) |  |

***Abbreviations:*** HCC, hepatocellular carcinoma; MF-ICC, mass-forming intrahepatic cholangiocarcinoma.

**Table S2.** Baseline characteristics of patients in the internal validation cohort

| **Variables** | **All (*n* = 101)** | **HCC (*n* = 76)** | **MF-ICC (*n* = 25)** | ***p* value** |
| --- | --- | --- | --- | --- |
| Age (year) | 54.95 ± 10.22 | 53.89 ± 10.54 | 58.16 ± 8.59 | 0.070 |
| Ki-67 (%) | 38.04 ± 20.25 | 36.61 ± 19.56 | 42.40 ± 22.04 | 0.184 |
| CK19, *n* (%) |  |  |  | <0.001 |
| -Negative | 74 (73.27) | 65 (85.53) | 9 (36.00) |  |
| -Positive | 27 (26.73) | 11 (14.47) | 16 (64.00) |  |
| Sex, *n* (%) |  |  |  | 1.000 |
| -Female | 26 (25.74) | 20 (26.32) | 6 (24.00) |  |
| -Male | 75 (74.26) | 56 (73.68) | 19 (76.00) |  |
| Tumor diameter, *n* (%) |  |  |  | <0.001 |
| -< 5 cm | 57 (56.44) | 54 (71.05) | 3 (12.00) |  |
| -≥ 5 cm | 44 (43.56) | 22 (28.95) | 22 (88.00) |  |
| Alpha-fetoprotein, *n* (%) |  |  |  | 0.040 |
| -< 400 ng/mL | 71 (70.30) | 58 (76.32) | 13 (52.00) |  |
| -≥ 400 ng/mL | 30 (29.70) | 18 (23.68) | 12 (48.00) |  |
| Hepatitis B surface antigen, *n* (%) |  |  |  | 0.869 |
| -Negative | 11 (10.89) | 9 (11.84) | 2 (8.00) |  |
| -Positive | 90 (89.11) | 67 (88.16) | 23 (92.00) |  |
| Cirrhosis, *n* (%) |  |  |  | 1.000 |
| -Absent | 30 (29.70) | 23 (30.26) | 7 (28.00) |  |
| -Present | 71 (70.30) | 53 (69.74) | 18 (72.00) |  |
| Intratumoral necrosis, *n* (%) |  |  |  | 0.109 |
| -Absent | 16 (15.84) | 9 (11.84) | 7 (28.00) |  |
| -Present | 85 (84.16) | 67 (88.16) | 18 (72.00) |  |
| Tumor margin, *n* (%) |  |  |  | 0.675 |
| -Smooth | 42 (41.58) | 33 (43.42) | 9 (36.00) |  |
| -Non-smooth | 59 (58.42) | 43 (56.58) | 16 (64.00) |  |
| Internal arteries, *n* (%) |  |  |  | 0.604 |
| -Absent | 50 (49.50) | 36 (47.37) | 14 (56.00) |  |
| -Present | 51 (50.50) | 40 (52.63) | 11 (44.00) |  |
| Peritumoral enhancement, *n* (%) |  |  |  | 0.603 |
| -Absent | 63 (62.38) | 49 (64.47) | 14 (56.00) |  |
| -Present | 38 (37.62) | 27 (35.53) | 11 (44.00) |  |
| Enhancing capsule, *n* (%) |  |  |  | 0.328 |
| -Absent | 46 (45.54) | 32 (42.11) | 14 (56.00) |  |
| -Present | 55 (54.46) | 44 (57.89) | 11 (44.00) |  |
| Arterial-phase rim enhancement, *n* (%) |  |  |  | <0.001 |
| -Absent | 71 (70.30) | 64 (84.21) | 7 (28.00) |  |
| -Present | 30 (29.70) | 12 (15.79) | 18 (72.00) |  |

***Abbreviations:*** HCC, hepatocellular carcinoma; MF-ICC, mass-forming intrahepatic cholangiocarcinoma.

**Table S3.** Baseline characteristics of patients in the external validation cohort

| **Variables** | **All (*n* = 50)** | **HCC (*n* = 40)** | **MF-ICC (*n* = 10)** | ***p* value** |
| --- | --- | --- | --- | --- |
| Age (year) | 54.44 ± 11.49 | 54.08 ± 10.07 | 55.90 ± 16.64 | 0.658 |
| Ki-67 (%) | 29.98 ± 20.21 | 27.27 ± 18.50 | 40.80 ± 24.01 | 0.083 |
| CK19, *n* (%) |  |  |  | 0.002 |
| -Negative | 35 (70.00) | 32 (80.00) | 3 (30.00) |  |
| -Positive | 15 (30.00) | 8 (20.00) | 7 (70.00) |  |
| Sex, *n* (%) |  |  |  | 0.082 |
| -Female | 12 (24.00) | 7 (17.50) | 5 (50.00) |  |
| -Male | 38 (76.00) | 33 (82.50) | 5 (50.00) |  |
| Tumor diameter, *n* (%) |  |  |  | <0.001 |
| -< 5 cm | 35 (70.00) | 33 (82.50) | 2 (20.00) |  |
| -≥ 5 cm | 15 (30.00) | 7 (17.50) | 8 (80.00) |  |
| Alpha-fetoprotein, *n* (%) |  |  |  | 0.782 |
| -< 400 ng/mL | 41 (82.00) | 32 (80.00) | 9 (90.00) |  |
| -≥ 400 ng/mL | 9 (18.00) | 8 (20.00) | 1 (10.00) |  |
| Hepatitis B surface antigen, *n* (%) |  |  |  | 0.128 |
| -Negative | 4 (8.00) | 2 (5.00) | 2 (20.00) |  |
| -Positive | 46 (92.00) | 38 (95.00) | 8 (80.00) |  |
| Cirrhosis, *n* (%) |  |  |  | <0.001 |
| -Absent | 12 (24.00) | 4 (10.00) | 8 (80.00) |  |
| -Present | 38 (76.00) | 36 (90.00) | 2 (20.00) |  |
| Intratumoral necrosis, *n* (%) |  |  |  | 0.659 |
| -Absent | 10 (20.00) | 9 (22.50) | 1 (10.00) |  |
| -Present | 40 (80.00) | 31 (77.50) | 9 (90.00) |  |
| Tumor margin, *n* (%) |  |  |  | 1.000 |
| -Smooth | 19 (38.00) | 15 (37.50) | 4 (40.00) |  |
| -Non-smooth | 31 (62.00) | 25 (62.50) | 6 (60.00) |  |
| Internal arteries, *n* (%) |  |  |  | 0.718 |
| -Absent | 30 (60.00) | 25 (62.50) | 5 (50.00) |  |
| -Present | 20 (40.00) | 15 (37.50) | 5 (50.00) |  |
| Peritumoral enhancement, *n* (%) |  |  |  | 1.000 |
| -Absent | 19 (38.00) | 15 (37.50) | 4 (40.00) |  |
| -Present | 31 (62.00) | 25 (62.50) | 6 (60.00) |  |
| Enhancing capsule, *n* (%) |  |  |  | 0.610 |
| -Absent | 31 (62.00) | 26 (65.00) | 5 (50.00) |  |
| -Present | 19 (38.00) | 14 (35.00) | 5 (50.00) |  |
| Arterial-phase rim enhancement, *n* (%) |  |  |  | 0.012 |
| -Absent | 30 (60.00) | 28 (70.00) | 2 (20.00) |  |
| -Present | 20 (40.00) | 12 (30.00) | 8 (80.00) |  |

***Abbreviations:*** HCC, hepatocellular carcinoma; MF-ICC, mass-forming intrahepatic cholangiocarcinoma.

**Table S4.** Confusion matrices of all models in the three cohorts

| **Models** | **TP** | **FN** | **TN** | **FP** | **F1 score** |
| --- | --- | --- | --- | --- | --- |
| *Internal training cohort* | | | | | |
| AP | 39 | 39 | 132 | 23 | 0.557 |
| PVP | 15 | 63 | 149 | 6 | 0.303 |
| DP | 9 | 69 | 155 | 0 | 0.207 |
| 2D | 37 | 41 | 144 | 11 | 0.587 |
| Ensemble | 33 | 45 | 142 | 13 | 0.532 |
| 2D+multi-channel+C | 61 | 17 | 138 | 17 | 0.782 |
| 2D+C | 56 | 22 | 140 | 15 | 0.752 |
| Multi-channel+C | 68 | 10 | 126 | 29 | 0.777 |
| Multi-channel | 57 | 21 | 117 | 38 | 0.659 |
| Clinical | 55 | 23 | 129 | 26 | 0.692 |
| *Internal validation cohort* | | | | | |
| AP | 18 | 7 | 58 | 18 | 0.590 |
| PVP | 17 | 8 | 59 | 17 | 0.576 |
| DP | 12 | 13 | 73 | 3 | 0.600 |
| 2D | 16 | 9 | 60 | 16 | 0.561 |
| Ensemble | 19 | 6 | 54 | 22 | 0.576 |
| 2D+multi-channel+C | 20 | 5 | 64 | 12 | 0.702 |
| 2D+C | 18 | 7 | 64 | 12 | 0.655 |
| Multi-channel+C | 23 | 2 | 55 | 21 | 0.667 |
| Multi-channel | 20 | 5 | 54 | 22 | 0.597 |
| Clinical | 18 | 7 | 64 | 12 | 0.655 |
| *External validation cohort* | | | | | |
| AP | 9 | 1 | 36 | 4 | 0.783 |
| PVP | 8 | 2 | 35 | 5 | 0.696 |
| DP | 3 | 7 | 40 | 0 | 0.462 |
| 2D | 8 | 2 | 37 | 3 | 0.762 |
| Ensemble | 9 | 1 | 35 | 5 | 0.750 |
| 2D+multi-channel+C | 8 | 2 | 36 | 4 | 0.727 |
| 2D+C | 7 | 3 | 34 | 6 | 0.609 |
| Multi-channel+C | 8 | 2 | 29 | 11 | 0.552 |
| Multi-channel | 7 | 3 | 29 | 11 | 0.500 |
| Clinical | 8 | 2 | 28 | 12 | 0.533 |

***Note.*** MF-ICC was treated as the positive class.

***Abbreviations:*** AP, arterial phase; PVP, portal venous phase; DP, delayed phase; 2D, prespecified stacking-based decision-level fusion model; C, clinical model; Ensemble, arithmetic mean of AP, PVP, and DP probabilities; TP, true positive; FN, false negative; TN, true negative; FP, false positive.

**Table S5.** Classification thresholds selected in the internal validation cohort

| **Models** | **AUC** | **Threshold** | **Sensitivity** | **Specificity** | **Youden index** |
| --- | --- | --- | --- | --- | --- |
| AP | 0.768 | 0.480 | 0.720 | 0.763 | 0.483 |
| PVP | 0.789 | 0.465 | 0.680 | 0.776 | 0.456 |
| DP | 0.764 | 0.501 | 0.480 | 0.961 | 0.441 |
| 2D | 0.754 | 0.476 | 0.640 | 0.789 | 0.429 |
| Ensemble | 0.779 | 0.448 | 0.760 | 0.711 | 0.471 |
| 2D+multi-channel+C | 0.887 | 0.419 | 0.800 | 0.842 | 0.642 |
| 2D+C | 0.863 | 0.448 | 0.720 | 0.842 | 0.562 |
| Multi-channel+C | 0.878 | 0.363 | 0.920 | 0.724 | 0.644 |
| Multi-channel | 0.814 | 0.357 | 0.800 | 0.711 | 0.511 |
| Clinical | 0.784 | 0.516 | 0.720 | 0.842 | 0.562 |

***Note.*** MF-ICC was treated as the positive class. Thresholds were selected by maximizing the Youden index in the internal validation cohort and were subsequently applied unchanged to the external validation cohort. Youden index = sensitivity + specificity − 1.

***Abbreviations:*** AP, arterial phase; PVP, portal venous phase; DP, delayed phase; 2D, prespecified stacking-based decision-level fusion model; C, clinical model; Ensemble, arithmetic mean of AP, PVP, and DP probabilities.

**Table S6.** Pairwise comparisons of AUCs among the six principal models

| **Model 1** | **Model 2** | **ΔAUC (95% CI)** | **Unadjusted *p* value** | **Bonferroni-adjusted *p* value** |
| --- | --- | --- | --- | --- |
| *Internal training cohort* | | | | |
| 2D | 2D+multi-channel+C | −0.064 (−0.106 to −0.023) | 0.002 | 0.033 |
| 2D | 2D+C | −0.065 (−0.112 to −0.018) | 0.006 | 0.095 |
| 2D | Multi-channel+C | −0.053 (−0.103 to −0.003) | 0.037 | 0.561 |
| 2D | Multi-channel | 0.037 (−0.016 to 0.089) | 0.172 | 1.000 |
| 2D | Clinical | 0.000 (−0.067 to 0.068) | 0.992 | 1.000 |
| 2D+multi-channel+C | 2D+C | −0.001 (−0.022 to 0.021) | 0.958 | 1.000 |
| 2D+multi-channel+C | Multi-channel+C | 0.011 (0.000 to 0.023) | 0.057 | 0.849 |
| 2D+multi-channel+C | Multi-channel | 0.101 (0.060–0.142) | <0.001 | <0.001 |
| 2D+multi-channel+C | Clinical | 0.065 (0.023–0.107) | 0.002 | 0.037 |
| 2D+C | Multi-channel+C | 0.012 (−0.012 to 0.036) | 0.330 | 1.000 |
| 2D+C | Multi-channel | 0.102 (0.044–0.160) | 0.001 | 0.009 |
| 2D+C | Clinical | 0.065 (0.037–0.094) | <0.001 | <0.001 |
| Multi-channel+C | Multi-channel | 0.089 (0.048–0.131) | <0.001 | <0.001 |
| Multi-channel+C | Clinical | 0.053 (0.015–0.092) | 0.007 | 0.104 |
| Multi-channel | Clinical | −0.036 (−0.109 to 0.037) | 0.329 | 1.000 |
| *Internal validation cohort* | | | | |
| 2D | 2D+multi-channel+C | −0.132 (−0.233 to −0.032) | 0.010 | 0.149 |
| 2D | 2D+C | −0.108 (−0.240 to 0.024) | 0.108 | 1.000 |
| 2D | Multi-channel+C | −0.124 (−0.247 to 0.000) | 0.049 | 0.737 |
| 2D | Multi-channel | −0.059 (−0.139 to 0.020) | 0.144 | 1.000 |
| 2D | Clinical | −0.029 (−0.208 to 0.150) | 0.749 | 1.000 |
| 2D+multi-channel+C | 2D+C | 0.024 (−0.032 to 0.080) | 0.395 | 1.000 |
| 2D+multi-channel+C | Multi-channel+C | 0.008 (−0.020 to 0.037) | 0.561 | 1.000 |
| 2D+multi-channel+C | Multi-channel | 0.073 (0.011–0.135) | 0.021 | 0.314 |
| 2D+multi-channel+C | Clinical | 0.103 (0.000 to 0.207) | 0.051 | 0.764 |
| 2D+C | Multi-channel+C | −0.016 (−0.061 to 0.029) | 0.494 | 1.000 |
| 2D+C | Multi-channel | 0.049 (−0.060 to 0.158) | 0.380 | 1.000 |
| 2D+C | Clinical | 0.079 (0.022–0.136) | 0.006 | 0.097 |
| Multi-channel+C | Multi-channel | 0.065 (−0.016 to 0.146) | 0.117 | 1.000 |
| Multi-channel+C | Clinical | 0.095 (0.011–0.179) | 0.027 | 0.412 |
| Multi-channel | Clinical | 0.030 (−0.122 to 0.182) | 0.699 | 1.000 |
| *External validation cohort* | | | | |
| 2D | 2D+ multi-channel+C | 0.012 (-0.079, 0.104) | 0.788 | 1.000 |
| 2D | 2D+C | 0.017 (-0.099, 0.134) | 0.768 | 1.000 |
| 2D | Multi-channel+C | 0.067 (-0.074, 0.209) | 0.351 | 1.000 |
| 2D | Multi-channel | 0.087 (-0.008, 0.183) | 0.073 | 1.000 |
| 2D | Clinical | 0.175 (-0.052, 0.402) | 0.131 | 1.000 |
| 2D+multi-channel+C | 2D+C | 0.005 (-0.053, 0.063) | 0.865 | 1.000 |
| 2D+multi-channel+C | Multi-channel+C | 0.055 (-0.011, 0.121) | 0.105 | 1.000 |
| 2D+multi-channel+C | Multi-channel | 0.075 (-0.017, 0.167) | 0.110 | 1.000 |
| 2D+multi-channel+C | Clinical | 0.163 (-0.008, 0.333) | 0.062 | 0.926 |
| 2D+C | Multi-channel+C | 0.050 (0.004, 0.096) | 0.032 | 0.480 |
| 2D+C | Multi-channel | 0.070 (-0.061, 0.201) | 0.294 | 1.000 |
| 2D+C | Clinical | 0.158 (0.036, 0.279) | 0.011 | 0.167 |
| Multi-channel+C | Multi-channel | 0.020 (-0.112, 0.152) | 0.766 | 1.000 |
| Multi-channel+C | Clinical | 0.108 (-0.010, 0.225) | 0.074 | 1.000 |
| Multi-channel | Clinical | 0.088 (-0.146, 0.321) | 0.463 | 1.000 |

***Note.*** ΔAUC was calculated as AUC (Model 1) – AUC (Model 2), with 95% confidence interval (CI) obtained from the correlated DeLong covariance estimate.

***Abbreviations:*** AP, arterial phase; PVP, portal venous phase; DP, delayed phase; 2D, prespecified stacking-based decision-level fusion model; C, clinical model; Ensemble, arithmetic mean of AP, PVP, and DP probabilities.

**Table S7.** Exploratory calibration measures for the six principal models

| **Models** | **Brier score (95% CI)** | **Calibration intercept (95% CI)** | **Calibration slope (95% CI)** |
| --- | --- | --- | --- |
| *Internal training cohort* | | | |
| 2D | 0.161 (0.147–0.176) | 0.721 (0.376–1.236) | 2.200 (1.704–2.918) |
| 2D+multi-channel+C | 0.130 (0.116–0.146) | 0.696 (0.375–1.183) | 2.590 (2.080–3.467) |
| 2D+C | 0.133 (0.120–0.147) | 0.757 (0.448–1.180) | 2.574 (2.071–3.342) |
| Multi-channel+C | 0.127 (0.110–0.145) | 0.262 (−0.002 to 0.641) | 1.884 (1.459–2.594) |
| Multi-channel | 0.164 (0.137–0.194) | −0.272 (−0.459 to −0.023) | 0.791 (0.578–1.085) |
| Clinical | 0.142 (0.117–0.169) | 0.000 (−0.198 to 0.266) | 1.000 (0.761–1.332) |
| *Internal validation cohort* | | | |
| 2D | 0.164 (0.139–0.190) | −0.346 (−0.675 to 0.035) | 1.443 (0.723–2.496) |
| 2D+multi-channel+C | 0.133 (0.114–0.152) | 0.115 (−0.304 to 0.795) | 2.693 (1.954–4.189) |
| 2D+C | 0.138 (0.118–0.160) | 0.025 (−0.331 to 0.537) | 2.284 (1.575–3.597) |
| Multi-channel+C | 0.129 (0.107–0.153) | −0.232 (−0.612 to 0.302) | 2.023 (1.368–3.498) |
| Multi-channel | 0.157 (0.121–0.196) | −0.719 (−0.984 to −0.338) | 0.851 (0.481–1.447) |
| Clinical | 0.157 (0.117–0.201) | −0.595 (−0.829 to −0.314) | 0.699 (0.348–1.271) |
| *External validation cohort* | | | |
| 2D | 0.111 (0.089, 0.136) | 0.207 (-0.847, 1.517) | 3.272 (2.057, 10.976) |
| 2D+multi-channel+C | 0.116 (0.091, 0.145) | -0.231 (-1.024, 1.064) | 2.709 (1.682, 6.192) |
| 2D+C | 0.113 (0.087, 0.141) | -0.132 (-0.818, 0.608) | 2.504 (1.624, 6.104) |
| Multi-channel+C | 0.130 (0.094, 0.172) | -0.711 (-1.269, -0.069) | 1.518 (0.780, 3.870) |
| Multi-channel | 0.158 (0.112, 0.215) | -1.138 (-1.562, -0.611) | 0.988 (0.460, 2.213) |
| Clinical | 0.154 (0.094, 0.216) | -1.096 (-1.692, -0.500) | 0.157 (-0.081, 1.835) |

***Abbreviations:*** AP, arterial phase; PVP, portal venous phase; DP, delayed phase; 2D, prespecified stacking-based decision-level fusion model; C, clinical model; Ensemble, arithmetic mean of AP, PVP, and DP probabilities; CI, confidence interval.
